# The prevalence and social determinants of fruit and vegetable consumption among adults in Kenya: a cross-sectional national population-based survey, 2015

**DOI:** 10.11604/pamj.2018.31.137.17039

**Published:** 2018-10-24

**Authors:** Supa Pengpid, Karl Peltzer

**Affiliations:** 1 ASEAN Institute for Health Development, Mahidol University, Salaya, Phutthamonthon, Nakhonpathom, Thailand; 2 Department of Research and Innovation, University of Limpopo, Turfloop, South Africa; 3 HIV/AIDS/STIs and TB Research Programme, Human Sciences Research Council, Pretoria, South Africa

**Keywords:** Fruit intake, vegetable intake, determinants, population survey, Kenya

## Abstract

**Introduction:**

Low fruit and vegetable consumption contributes significantly to the burden of disease. The study aimed to assess the prevalence and correlates of fruit and vegetable (FAV) consumption among adults in a national survey in Kenya.

**Methods:**

A national cross-sectional study based on a stratified cluster random sampling was conducted in 2015. The total sample included 4479 individuals 18-69 years, (females = 60.0%; median age 38.0 years, Inter Quartile Range 23) from Kenya. Sociodemographics, health risk behaviour and anthropometric data were collected using the WHO-STEPS questionnaire.

**Results:**

On average, participants had 0.78 servings of fruits a day, 1.31 servings of vegetables a day, and 2.09 servings of FAV. Only 12.4% of respondents had two or more servings of fruit a day, 7.4% had three or more servings of vegetables a day and 94.0% had less than five servings of FAV a day. In adjusted logistic regression analysis, higher education (Odds Ratio=OR: 1.68, Confidence Interval = CI: 1.18, 2.39), greater wealth (OR: 1.61, CI: 1.04, 2.48), and being a Kikuyu (OR: 2.17, CI: 1.46, 3.23) or Luo (OR: 1.58, CI: 1.05, 2.37) were associated with two or more servings of fruits daily. Urban residence (OR: 0.44, CI: 0.23, 0.82) and being male (OR: 0.72, CI: 0.53, 0.98) decreased the odds, and older age (OR: 1.68, CI: 1.05, 2.69) and being Luo (OR: 2.84, CI: 1.53, 5.27) increased the odds of having three or more servings of vegetables daily. Being male (OR: 0.71, CI: 0.52, 0.99) and being Luo (OR: 0.40, CI: 0.23, 0.70) decreased the odds and urban residence (OR: 2.50, CI: 1.27, 4.96) increased the odds of inadequate (< five servings) FAV consumption.

**Conclusion:**

A high prevalence of inadequate FAV consumption was found, and risk factors identified, such as being female, lower education, urban residence, and not being Luo, that may help in guiding strategies to increase FAV consumption.

## Introduction

Globally, 2.8% of deaths (mainly ischaemic heart disease, stroke and gastrointestinal cancer death) can be attributed to low fruit and vegetable (FAV) consumption [1]. Based on the health benefits of fruits and vegetables consumption, the World Health Organization [2] recommended the consumption of at least 400 g (5 servings) of FAV per day, and a further increase to 600 g per day could further significantly reduce the worldwide burden of disease [3]. In a study in 52 mainly low- and middle-income countries, 77.6% of men and 78.4% of women consumed less than five daily servings of FAV [4]. In the 2004 Kenya World Health Survey, the prevalence of insufficient (< five servings) FAV consumption was 86.7% [5]. There is a lack of recent national data on the prevalence and correlates of FAV consumption in Kenya. In countries in the African region, the prevalence of inadequate (< five servings) FAV consumption was 98.5% in Ethiopia [6], 95.8% in Mozambique [7] and 82% in Tanzania [8]. The median daily number of servings of fruits was two in Tanzania, while the average daily number of servings of vegetables was two in Tanzania [8]. In a study in 18 countries, including South Africa and Zimbabwe in Africa, the mean daily consumption of FAV was 2.14 servings in low-income countries, 3.17 servings in lower-middle-income countries and 4.31 servings in upper-middle-income countries [9]. In Mozambique, 17.8% of the participants met the recommended daily fruit consumption rate (≥ 2 servings) and 18.7% the recommended daily vegetable consumption rate (≥ 3 servings) [7]. Various factors have been found associated with low FAV consumption, including sociodemographic and health factors. Sociodemographic factors associated with low FAV consumption include, older age [4, 10, 11], male sex [6, 10, 12], lower education [10, 11], lower wealth [4, 6, 10, 11], and residing in urban areas [7]. Health factors associated with low FAV consumption include, high physical activity [10], physical inactivity [12, 13], not having overweight [10], smoking [14], alcohol intake [15], and binge drinking [12, 13]. The aim of this study was to assess the prevalence and social determinants of FAV consumption among adults in national survey in Kenya.

## Methods

**Data, study design and participants:** A multi-stage cluster sampling method was used to select adults aged 18 to 69 years for the Kenya STEPS Survey (April to June 2015), details of sampling methodology can be found in Ministry of Health [16]. The sample included 4479 participants with complete FAV measurements. The response rate for STEP 1 (questionnaire) was 95%, and STEP 2 (anthropometric measurements) 99% [16]. The Kenya Ministry of Health ethics committee approved the study protocol and participants provided written informed consent prior to the study.

Measures: Following WHO STEPS methodology [17] three steps were followed: “step 1, questionnaire interview; step 2, anthropometric measurements; and step 3, biochemistry tests. For step 1, handheld devices loaded with eSTEPS software and WHO STEPS questionnaire were used by trained data collectors at respondents' residences. Questions included tobacco use, alcohol consumption, dietary habits, the amount of physical activity, and sociodemographic profiles. Show cards on different types of tobacco products, alcohol, physical activities, and servings of FAV were used to facilitate understanding of the questions by the respondents" [17]. Daily *FAV intake* were calculated from the number of servings of FAV consumed per day in a typical week. Inadequate FAV consumption was defined as less than five servings a day [**2**] and recommended fruit consumption two or more servings and recommended vegetable consumption three or more servings a day [18].

*Physical activity* level was calculated from the duration of moderate and vigorous physical activities (at work, transport and recreation) in a typical day and week. Physical activity levels were classified into low, moderate and high, as per WHO Global Physical Activity Questionnaire [19]. *Current tobacco* use was measured with two questions, “Do you currently smoke any tobacco products, such as cigarettes, hand-rolled, cigars, water pipes/shisha or pipes/kiko?” and “Do you currently use any smokeless tobacco products such as snuff, chewing tobacco, kuber..pan?” (Yes, No) (16, p.5-1-6). *Past month binge drinking* was assessed by asking participants how many times they had six or more standard alcoholic drinks in a single drinking occasion during the past 30 days [16]. Step 2 involved taking height, weight, and waist circumference measurements. *Body Mass Index*(BMI) was measured from scientific body height and weight measures, and overweight or obesity was classified as 25+ kg/m^2^ [20]. Central obesity was defined as “Waist Circumference (WC) > 102 cm in men and > 88 cm in women.” [21]

**Data analysis:** Post stratification adjustments were done to align with the population projections according to age-sex categories [16]. Descriptive statistics on frequency, weighted prevalence and 95% confidence intervals (CI) was performed for sociodemographic, health and FAV variables. Differences in proportions and means were calculated with parametric and non-parametric tests. Logistic regression was conducted to assess associations between sociodemographic factors, health variables and three outcomes: adequate fruit consumption, 2. adequate vegetable consumption and 3. Inadequate FAV consumption. All analyses were adjusted for the multi-stage sample design and conducted with STATA software version 13.0 (Stata Corporation, College Station, TX, USA).

## Results

**Sample characteristics and intake of fruit, vegetables and fruit and vegetables categories:** The total sample included 4479 individuals 18-69 years, (females = 60.0%; median age 38.0 years, Inter Quartile Range 23) from Kenya. More than half of the participants had at least completed primary education (58.7%), 14.9% were Kikuyu by ethnic group, and 51.4% resided in rural areas. Almost one in ten (9.0%) of participants had general obesity, 12.8% central obesity, 89.1% had adequate physical activity, 13.3% were currently tobacco users and 13.5% were past month binge drinkers. Only 15.3% of participants consumed fruits daily, and 50.9% had vegetables daily. The mean number of days consuming fruits per week was 2.52 and the mean number of days consuming vegetables per week was 4.96. On average, participants had 0.78 servings of fruits a day, 1.31 servings of vegetables a day, and 2.09 servings of FAV per day. Only 12.4% of respondents had two or more servings of fruit a day, 7.4% had three or more servings of vegetables a day and 94.0% had less than five servings of FAV a day. In bivariate analyses, fruit servings per day increased with being male, education, greater wealth, urban residence, having general and central obesity, and being a Kikuyu or Luo by ethnic group and decreased with age, current tobacco use and low physical activity. Vegetables servings increased with age, education, being a Kikuyu or Luo by ethnic group and central obesity and decreased with greater wealth and low physical activity. FAV consumption increased with age, education, greater wealth, being a Kikuyu or Luo by ethnic group, and central obesity and decreased with low physical activity and current tobacco use (Table 1).

**Table 1:** Daily intake of fruit, vegetables and fruit and vegetables of 4479 adult participants by socio-demographic and health variables, Kenya national STEPS survey 2015

Variable	Sample	Fruit servings/day	Vegetable servings/day	Fruit and vegetable servings/day	Fruits (≥2 servings)/Day	Vegetables (≥3 servings)/Day	Fruit and vegetable (<5 servings)/Day
	N (%)	M (SD)	M (SD)	M (SD)	%	%	%
**Socio-demographics**							
**All Age in years**	4479	0.78 (1.2)	1.31 (1.1)	2.09 (1.7)	12.4	7.4	94.0
18-29	1494 (33.4)	0.84 (1.2)	1.18 (1.0)	2.02 (1.6)	13.6	4.8	94.4
30-44	1702 (38.0)	0.75 (1.3)	1.39 (1.1)	2.14 (1.8)	10.6	9.4	93.9
45-59	869 (19.4)	0.73 (1.2)	1.43 (1.0)	2.17 (1.6)	12.3	10.3	94.3
60-69	414 (9.2)	0.68 (1.2)*	1.48 (1.2)*	2.15 (1.6)	12.2	9.6*	90.5
**Sex**							
Female	2690 (60.1)	0.73 (1.1)	1.33 (1.1)	2.06 (1.7)	11.8	8.3	94.8
Male	1789 (39.8)	0.84 (1.2)*	1.28 (1.1)	2.12 (1.7)	12.9	6.5	93.2*
**Education**							
Primary school complete or more	2631 (58.7)	0.89 (1.1)	1.36 (1.0)	2.25 (1.6)	15.1	7.1	93.8
No/ Primary school incomplete	1848 (41.3)	0.58 (1.2)*	1.21 (1.2)*	1.79 (1.8)*	7.5*	8.0	94.4
**Wealth quintile**							
Poorest/Second	1792 (39.8)	0.62 (1.2)	1.29 (1.1)	1.91 (1.8)	8.7	8.7	94.0
Middle	896 (18.3)	0.84 (1.3)	1.50 (1.1)	2.34 (1.9)	11.5	10.4	92.5
Fourth/Richest	1791 (41.9)	0.91 (1.0)*	1.24 (1.0)*	2.15 (1.5)*	16.2*	4.9	94.7
**Residence**							
Rural	2300 (51.4)	0.75 (1.3)	1.37 (1.2)	2.13 (1.8)	11.3	9.3	92.8
Urban	2179 (48.6)	0.83 (1.0)*	1.20 (0.9)	2.03 (1.4)*	14.0	4.3*	96.0*
**Ethnic group**							
Kikuyu	666 (14.9)	1.07 (1.2)	1.33 (0.9)	2.40 (1.5)	22.0	8.2	93.2
Luo	434 (10.1)	0.99 (1.6)	1.65 (1.4)	2.64 (2.1)	15.2	13.5	89.2
Other	3379 (74.9)	0.70 (1.1)*	1.26 (1.0)*	1.95 (1.6)*	10.1*	6.4*	94.8*
**Health variables**							
Less than BMI <30 kg/m^2^	3824 (91.0)	0.76 (1.2)	1.30 (1.1)	2.06 (1.7)	11.6	7.2	94.1
≥30 kg/m^2^ (Obesity)	444 (9.0)	0.99 (1.2)*	1.34 (1.0)	2.34 (1.8)	20.0*	7.7	92.4
No central obesity	3568 (87.2)	0.77 (1.2)	1.29 (1.1)	2.05 (1.7)	11.6	7.0	94.0
Central obesity	698 (12.8)	0.89 (1.1)*	1.43 (1.1)*	2.33 (1.7)*	17.7*	8.6	93.1
Physical activity (Moderate/High)	3854 (89.1)	1.65 (1.6)	1.72 (1.1)	3.37 (2.2)	12.7	7.7	93.9
Physical activity (Low)	529 (10.9)	1.27 (1.2)*	1.46 (1.0)*	2.73 (1.8)*	8.8	5.9	96.3*
No current tobacco use	3930 (86.7)	0.80 (1.2)	1.32 (1.1)	2.11 (1.7)	12.9	7.6	93.9
Current tobacco use	549 (13.3)	0.68 (1.2)*	1.25 (1.1)	1.93 (1.7)*	8.9	6.3	94.6
No past month binge drinking	3997 (86.5)	0.77 (1.2)	1.31 (1.1)	2.08 (1.7)	12.4	7.6	94.0
Past month binge drinking	482 (13.5)	0.87 (1.2)	1.29 (1.0)	2.16 (1.6)	12.2	6.4	94.0

* <0.01

**Associations with fruit and vegetable consumption:** in adjusted logistic regression analysis, higher education (Odds Ratio = OR: 1.68, Confidence Interval=CI: 1.18, 2.39), greater wealth (OR: 1.61, CI: 1.04, 2.48), and being a Kikuyu (OR: 2.17, CI: 1.46, 3.23) or Luo (OR: 1.58, CI: 1.05, 2.37) were associated with two or more servings of fruits daily. Urban residence (OR: 0.44, CI: 0.23, 0.82) and being male (OR: 0.72, CI: 0.53, 0.98) decreased the odds, and older age (OR: 1.68, CI: 1.05, 2.69) and being Luo (OR: 2.84, CI: 1.53, 5.27) increased the odds of having three or more servings of vegetables daily. Being male (OR: 0.71, CI: 0.52, 0.99) and being Luo (OR: 0.40, CI: 0.23, 0.70) decreased the odds and urban residence (OR: 2.50, CI: 1.27, 4.96) increased the odds of inadequate (< five servings) FAV consumption (Table 2).

**Table 2:** Associations of independent variables with fruit (≥ 2 servings), vegetable (≥ 3 servings) and inadequate fruit and vegetable (< 5 servings) consumption

Variable	Fruits (≥ 2 servings) (≥ 3 servings)	Vegetables	Fruit and vegetable (< 5 servings)
	AOR (95% CI)	AOR (95% CI)	AOR (95% CI)
**Socio-demographics**			
**Age in years**			
18-29	1 (Reference)	1 (Reference)	1 (Reference)
30-44	0.75 (0.55, 1.02)	1.69 (1.15, 2.46)**	1.00 (0.70, 1.42)
45-59	0.95 (0.69, 1.30)	1.87 (1.27, 2.77)**	1.09 (0.72, 1.67)
60-69	1.01 (0.62, 1.68)	1.68 (1.05, 2.69)*	0.66 (0.41, 1.06)
**Sex**			
Female	1 (Reference)	1 (Reference)	1 (Reference)
Male	1.16 (0.87, 1.55)	0.72 (0.53, 0.98)*	0.71 (0.52, 0.99)*
**Education**			
No/Primary school incomplete	1 (Reference)	1 (Reference)	1 (Reference)
Primary school complete or more	1.68 (1.18, 2.39)**	1.10 (0.71, 1.71)	0.91 (0.55, 1.50)
**Wealth quintile**			
Poorest/Second	1 (Reference)	1 (Reference)	1 (Reference)
Middle	1.07 (0.77, 1.49)	1.33 (0.90, 1.96)	0.73 (0.48, 1.12)
Fourth/Richest	1.61 (1.04, 2.48)*	0.79 (0.36, 1.72)	0.74 (0.36, 1.49)
**Ethnic group**			
Other	1 (Reference)	1 (Reference)	1 (Reference)
Kikuyu	2.17 (1.46, 3.23)***	1.49 (0.70, 3.15)	0.76 (0.39, 1.47)
Luo	1.58 (1.05, 2.37)*	2.84 (1.53, 5.27)***	0.40 (0.23, 0.70)**
Urban residence (base=rural residence)	0.78 (0.53, 1.13)	0.44 (0.23, 0.82)**	2.50 (1.27, 4.96)**
**Health variables**			
Body Mass Index ≥30 kg/m^2^(Obesity)	1.48 (0.98, 2.24)	0.95 (0.59, 1.53)	0.80 (0.42, 1.51)
Central obesity (base= no)	1.28 (0.80, 2.04)	1.08 (0.68, 1.73)	0.80 (0.49, 1.30)
Physical activity (Low) (base=Moderate/High)	0.65 (0.42, 1.03)	0.98 (0.58, 1.67)	1.10 (0.67, 1.82)
Current tobacco use (base=no)	0.75 (0.44, 1.29)	0.89 (0.54, 1.47)	1.11 (0.58, 1.90)
Binge drinking (base=no)	1.10 (0.70, 1.72)	1.16 (0.71, 1.90)	1.00 (0.58, 1.70)

AOR=Adjusted Odds Ratio; CI=Confidence Interval

*** P<.001

** P<.01

* P<.05

## Discussion

In this nationally representative population-based survey on FAV consumption in Kenya, the age-adjusted prevalence of inadequate (< 5 servings) FAV consumption in adults (18-69 years) was 94.0% in 2015, which is higher than in the 2004 Kenya World Health Survey (86.7%) [5], in Tanzania (82%) [8], and a global (52 countries) average (77.6% of men and 78.4% of women) [4], but similar to Ethiopia (98.5%) [6] and Mozambique (95.8%) [7]. This finding seems to indicate a deterioration in FAV consumption from 2004 to 2015 in Kenya, which may be attributed a nutrition transition dominated by bought and processed foods reducing FAV consumption [22]. This finding calls for urgent health promotion programming to increase FAV consumption in Kenya. In this study only 15.3% of participants consumed fruits daily, and 50.9% had vegetables daily, which is lower than in a national survey in Thailand (36.5% consumed fruit and 68.0% vegetables daily) [11]. On average, participants had 0.78 servings of fruits a day and 1.31 servings of vegetables a day in this study, which is lower than in Tanzania (2 servings of fruit and 2 servings of vegetables a day) [8]. The average daily number of servings of FAV consumed was 2.09 servings in this study in Kenya (being a lower-middle income country), which compares with 2.14 servings in low-income countries but is much lower than 3.2 servings in lower-middle income countries of the PURE study [9]. Only 12.4% of respondents met the recommended daily fruit consumption rate (≥ 2 servings), which was lower than in Mozambique (17.8%) [7], and only 7.4% met the recommended daily vegetable consumption rate (≥ 3 servings), which was also lower than in Mozambique (18.7%) [7].

This would mean more effort is needed to increase the frequency of both FAV intake in order to reach recommended levels of FAV consumption. While some studies found a decline of inadequate FAV consumption with age [4, 10, 11], this study did not find significant age differences. In fact, there was an increase in vegetable consumption with age, meaning that in particular young people lacked vegetable consumption, which should be targeted in the promotion of increase in vegetable consumption. Some previous studies found a preponderance of inadequate FAV consumption among men [6, 10, 12], this study found a higher insufficient FAV consumption among women than men. This study found in agreement with previous studies [4, 6, 10, 11] that higher socioeconomic status was associated with adequate fruit consumption. Greater educational attainment may be accompanied with better dietary knowledge and skills to implement eating fruits [10]. In addition, greater wealth may lead to better affordability to purchase fruits. In this study, urban residence increased the odds for inadequate FAV consumption, which was also found in a national study in Mozambique [7]. It is possible that availability and accessibility of a variety of FAV is lower in urban than in rural areas. Moreover, a stronger nutrition transition with a change to more processed foods may be taken place in urban compared with rural areas in Kenya [22]. Compared to other ethnic groups, belonging to the Luo ethnic group decreased the odds for inadequate FAV consumption and being a Kikuyu increased the odds for fruit consumption in this study. The Luo and Kikuyu are among the most educated ethnic groups in Kenya [23], which may contribute via better knowledge and skills to higher FAV consumption. Unlike some previous studies [12-14], this study did not find any association between tobacco use, binge drinking and inadequate FAV. Likewise, in adjusted analysis physical inactivity and body weight status were in this study not significantly associated with inadequate FAV. While some other previous studies [10, 12, 13] found an association with inadequate FAV.

**Study limitations:** Apart from anthropometric measurements, a study limitation was that all the other information assessed in this analysis was based on self-reporting. It is possible that certain behaviours were over or under reported. Further, it was a cross-sectional study and causal relationships between independent and dependent variables cannot be established. Certain variables that represent barriers or facilitators such as cost and access were not assessed in this study and should be included in future studies.

## Conclusion

The study found a high prevalence of inadequate FAV consumption in a representative sample of the general adult population in Kenya. Relative to other studies, vegetable consumption was particularly low. Several risk factors, such as being female, lower education, urban residence, and not being Luo were identified to better target an increase in FAV consumption in Kenya.

### What is known about this topic

- In the 2004 Kenya World Health Survey, the prevalence of insufficient (< five servings) fruit and vegetable consumption was 86.7%.

### What this study adds

- The current (2015) study showed that the prevalence of insufficient fruit and vegetable consumption was 94.0% in Kenya;
- Several risk factors, such as being female, lower education, urban residence, and not being Luo were identified for insufficient fruit and vegetable consumption;
- Very high insufficient fruit and vegetable consumption calls for urgent health promotion programming to increase fruit and vegetable consumption in Kenya.

## Competing interests

The authors declare no competing interests.
